# Increased Pituitary Fluorine-18-Fluorodeoxyglucose Uptake in Patients with Differentiated Thyroid Cancer in Hypothyroidism versus under Recombinant Human Thyroid-Stimulating Hormone Stimulation

**DOI:** 10.3390/cancers16071382

**Published:** 2024-03-31

**Authors:** Xinyi Shi, Ilaria Giordani, Marie Nicod Lalonde, Gerasimos P. Sykiotis

**Affiliations:** 1 Service of Endocrinology, Diabetology and Metabolism, Lausanne University Hospital, University of Lausanne, 1011 Lausanne, Switzerland; xinyi.shi@chuv.ch (X.S.); ilaria.giordani@chuv.ch (I.G.); 2 Service of Nuclear Medicine and Molecular Imaging, Lausanne University Hospital, University of Lausanne, 1011 Lausanne, Switzerland; marie.nicod-lalonde@chuv.ch

**Keywords:** ^18^F-FDG PET/CT, pituitary hypermetabolism, thyroid carcinoma, hypothyroidism, rhTSH

## Abstract

**Simple Summary:**

The incidental pituitary hypermetabolism on ^18^F-FDG PET/CT should be further evaluated for discriminating between pathologic and physiologic uptake, but a recent study suggests that pituitary hypermetabolism is common in patients with differentiated thyroid carcinoma (DTC) undergoing thyroid hormone withdrawal (THW). The aim of this retrospective study was to compare pituitary metabolism in patients with DTC undergoing ^18^F-FDG PET/CT under THW versus recombinant human thyroid-stimulating hormone (rhTSH) stimulation. We confirmed a higher pituitary SUVmax and SUVratio with a higher prevalence of pituitary hypermetabolism in the THW group compared to the rhTSH group. A positive correlation between serum TSH levels and pituitary SUVmax was observed only in the THW group. The present findings support the hypothesis that pituitary hypermetabolism on ^18^F-FDG PET/CT in patients with DTC undergoing THW is a common physiological response to hypothyroidism. Awareness of this physiological hypermetabolism is important to avoid potential pitfalls in image interpretation that could trigger unnecessary investigations.

**Abstract:**

Background: ^18^F-FDG PET/CT is performed for the assessment of radioactive iodine non-avid disease in patients with DTC. In patients prepared by THW, increased pituitary uptake of ^18^F-FDG in the absence of pituitary disease may reflect the physiological activation of pituitary thyrotroph cells by hypothyroidism. This study aimed to compare pituitary ^18^F-FDG uptake in patients with DTC under THW vs. rhTSH stimulation. Methods: A total of 57 patients with DTC undergoing ^18^F-FDG PET/CT (40 under THW and 17 under rhTSH stimulation) were retrospectively analyzed. Pituitary metabolism was expressed as maximum standardized uptake value (SUVmax) and as SUVratio using the right cerebellum as reference. Results: Pituitary hypermetabolism (SUVmax ≥ 4.1) was present in more patients in the THW group compared to the rhTSH group (62.5% vs. 23.5%; *p* = 0.01). Pituitary metabolism was significantly higher in the THW group compared to the rhTSH group, as assessed by either SUVmax (mean ± SD: 4.61 ± 1.22, 95%CI: 4.22–5.00 vs. 3.34 ± 0.86, 95%CI: 2.9–3.8; *p* < 0.001) or SUVratio (0.52 ± 0.11, 95%CI: 0.49–0.56 vs. 0.42 ± 0.07, 95%CI: 0.38–0.46; *p* < 0.001). Serum TSH levels correlated positively with SUVmax (r = 0.41, *p* < 0.01) and SUVratio (r = 0.44, *p* < 0.01) in the THW group only. Conclusions: The present findings support the hypothesis that pituitary hypermetabolism on ^18^F-FDG PET/CT in patients with DTC undergoing THW is a common physiological response to hypothyroidism. Awareness of this physiological hypermetabolism is important to avoid potential pitfalls in image interpretation.

## 1. Introduction

In patients with differentiated thyroid carcinoma (DTC), fluorine-18 fluorodeoxyglucose positron emission tomography/computed tomography (^18^F-FDG PET/CT) imaging may be performed when indicated for assessment of residual or recurrent disease, especially radioactive iodine (RAI) non-avid disease, at the time of RAI therapy or at other times, such as when thyroglobulin levels rise after RAI therapy [1]. To increase radioiodine uptake by DTC cells, RAI therapy requires high levels of TSH, which may be obtained by either thyroid hormone withdrawal (THW) or the administration of recombinant human thyroid-stimulating hormone (rhTSH, Thyrogen^®^). The clinical advantage of rhTSH is that patients continue thyroid hormone substitution, thereby avoiding symptoms of hypothyroidism [2,3,4]. The rationale for performing ^18^F-FDG PET/CT at the time of RAI therapy is that the high TSH levels may increase the detection rate of metastatic DTC lesions; similarly, outside RAI therapy, rhTSH may be used to potentially increase the sensitivity of ^18^F-FDG PET/CT to detect low-volume disease [5].

Previous studies have demonstrated that thyroid function can impact the metabolic activity of various tissues, as evidenced in ^18^F-FDG PET/CT scans [6]. Under physiological conditions, the pituitary gland shows little uptake of ^18^F-FDG; incidental findings of pituitary hypermetabolism are unusual, with a reported frequency of 0.8% [7,8,9,10]. Therefore, pituitary incidentalomas identified on the ^18^F-FDG PET/CT merit further diagnostic workup to discriminate between clinically significant pathologic pituitary uptake and nonspecific physiologic pituitary uptake; the most common disease was a primary pituitary tumor, metastatic malignancy, Langerhans cell histiocytosis, and inflammatory hypophysitis [8,9]. Few studies have addressed the clinical relevance of incidental pituitary hypermetabolism in patients with DTC. A recent study found that pituitary ^18^F-FDG uptake among 215 patients with DTC undergoing THW was increased compared to 215 healthy subjects, and it correlated positively with serum TSH levels [11]. The available patient data showed no evidence of pituitary disease, suggesting that the increased uptake most likely represents a physiological consequence of hypothyroidism, possibly linked to the increased activity of pituitary thyrotroph cells during THW. However, this hypothesis remains to be confirmed by direct comparison to a group of patients with DTC undergoing ^18^F-FDG PET/CT via rhTSH stimulation. Therefore, in this study, we examined retrospective data from a cohort of patients with DTC who underwent ^18^F-FDG PET/CT via either THW or rhTSH stimulation using the same instrument and imaging protocol. We documented the frequency of pituitary hypermetabolism in ^18^F-FDG PET/CT via THW or rhTSH, investigated its potential clinical significance, and examined the correlation of pituitary ^18^F-FDG uptake with serum TSH levels.

## 2. Materials and Methods

### 2.1. Patients

This was a retrospective cohort study of patients treated for DTC at Lausanne University Hospital. Patients were included if they (i) had a total thyroidectomy with a diagnosis of DTC; (ii) had undergone ^18^F-FDG PET/CT via THW or rhTSH stimulation on the same PET/CT instrument (cPET, GE Discovery 690) between 1 January 2015 and 1 October 2022, whether it was before, during, or after any RAI treatment; (iii) were ≥18 years old at the time of imaging; (iv) had no known pituitary disease; and (v) had signed the hospital’s general informed consent form for reuse of health data in research. Based on a Wilcoxon–Mann–Whitney test with a large, expected effect size (d = 0.8) and an allocation ratio of 2:1 between the THW and rhTSH groups, respectively, a total sample size of 48 was calculated in order to have 80% power to detect a difference at a *p*-value of <0.05. A total of 57 patients imaged between 2015 and 2022 were included; 40 had been imaged under THW and 17 under rhTSH stimulation. All patients with THW had undergone thyroid function tests within 7 days of the PET/CT scan. Tumors were staged based on the Union for International Cancer Control (UICC) TNM Classification of Malignant Tumors 8th edition. Histology reports employing previous classifications were re-staged as per the 8th edition, according to the respective tumor characteristics. The study was approved by the Cantonal Ethics Commission for Research on Human Beings of the Canton of Vaud (CER-VD authorization #2022-01590).

### 2.2. ^18^F-FDG PET/CT Acquisition, Reconstruction, and Data Analysis

According to the hospital’s standard clinical protocol for oncologic acquisitions in a conventional PET instrument, each patient fasted for ≥6 h before the PET/CT scan to ensure a glucose concentration <11 mmol/L. Images were acquired 1 h after i.v. injection of 3.5 MBq/kg of ^18^F-FDG. The patient’s head was stabilized by head restraints. Acquisition for the head and neck region took 3 min per bed position. The imaging reconstruction was performed as previously described [12]. The glycemia, weight, body mass index, activity of ^18^F-FDG administered, and the time lapse between ^18^F-FDG injection and image acquisition were all documented.

**Figure 1:**
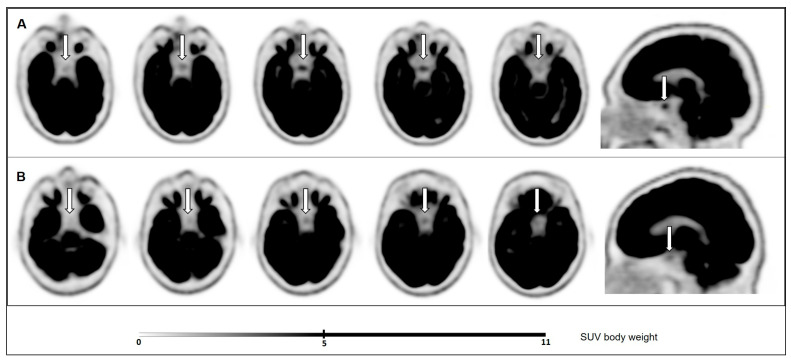
Pituitary gland (white arrows) ^18^F-FDG uptake signal in axial and sagittal planes in (**A**) a representative patient under THW (SUVmax = 4.4, SUVratio = 0.54); and (**B**) a representative patient under rhTSH stimulation (SUVmax = 3, SUVratio = 0.34).

For each patient, the pituitary metabolic activity was quantified by manually measuring the standardized uptake value (SUV). The SUVmax of the pituitary was measured by defining a region of interest encompassing the gland, and the SUVratio was calculated by dividing the pituitary SUVmax by the SUVmean of the right cerebellum as a reference region (13–17 mm). Pituitary SUV was also visualized using a readout scale of 0–5 g/mL, taking body weight into account (Figure 1). Pituitary hypermetabolism was determined by using a cut-off value of SUVmax ≥4.1, as customary [7,10,12].

### 2.3. Thyroid Function Tests and Glucose Measurements

Serum TSH levels (reference range: 0.270–4.20 mIU/L) and free thyroxin (fT4) levels (reference range: 12–22 pmol/L) were measured at the hospital’s laboratory using an electrochemiluminescence immunoassay (ECLIA) on a Cobas e801 machine (Roche Diagnostics, Basel, Switzerland) using samples obtained within 7 days of the PET/CT scans. Capillary glucose levels were measured with a portable glucose meter.

### 2.4. Statistical Analyses

Scale variables were expressed as mean ±standard deviation (SD) or mean ±95% confidence interval (CI). SUV values were compared between the THW and rhTSH groups using the Wilcoxon-Mann–Whitney test. Categorical variables were compared using the chi-square test. The correlation between serum TSH levels and pituitary SUVmax or SUVratio values was tested in the THW group and in the rhTSH group using Spearman’s rank test. In female patients, the effects of menopausal status and RAI preparation method on pituitary metabolism were tested by two-way ANOVA. GraphPad Prism 9 was used to perform the analyses and prepare the graphs.

## 3. Results

### 3.1. Patient Characteristics

All data associated with this study are included in Supplementary Table S1, available online [https://zenodo.org/records/10782396 (accessed on 5 March 2024)]. Patient characteristics are listed in Table 1. In the THW group, all 40 patients were overtly hypothyroid, with serum-free T4 levels <6 pmol/L. In the rhTSH group, of the 10/17 patients with available serum-free T4 levels and 11/17 with available serum TSH levels, 5 had free T4 levels between 6 and 12 pmol/L, and 8 had levels ≥12 pmol/L; none of the patients in this group reported symptoms of hypothyroidism (patients without TSH/free T4 values had ^18^F-FDG PET/CT imaging without RAI treatment). All available serum TSH levels were >30 mIU/L, which is generally adopted as a threshold for RAI therapy or diagnostic testing [13]. There were no statistically significant differences in weight, body mass index, ^18^F-FDG activity administered, glycemia, or time post-injection between the two groups. In the THW group, patients were younger (43.26 ± 16.97 vs. 54.68 ± 15.36, *p* = 0.02), and there were more female patients (85% vs. 41%, *p* < 0.01) than in the rhTSH group.

There were no statistically significant differences between the THW and rhTSH groups regarding the distribution of tumor histologies or the perceived indications for performing ^18^F-FDG PET/CT imaging (Table 1). Of note, minimal extrathyroidal extension (ETE) in the perithyroidal adipose tissue, previously staged as T3 disease, has been removed in the UICC 8th edition. Seven patients in the THW group and five patients in the rhTSH group had minimal ETE. It is interesting to note that despite this reclassification, only 1 patient would not meet current criteria for RAI treatment, as the other 11 patients (6 in the THW group and 5 in the rhTSH group) all had lymph node metastases warranting RAI.

### 3.2. Pituitary Metabolic Activity on ^18^F-FDG PET/CT and Correlation with Serum TSH Levels

In the THW group, 25/40 patients presented pituitary hypermetabolism, defined as SUVmax ≥ 4.1, which was significantly higher compared to the 4/17 patients in the rhTSH group (62.5% vs. 23.5%, *p* = 0.01). Of the total 29 patients with pituitary hypermetabolism, only 1 was further investigated and had a normal pituitary upon magnetic resonance imaging. The available clinical follow-up data on these patients did not show any evidence of pituitary metastasis or other pituitary pathology.

**Figure 2:**
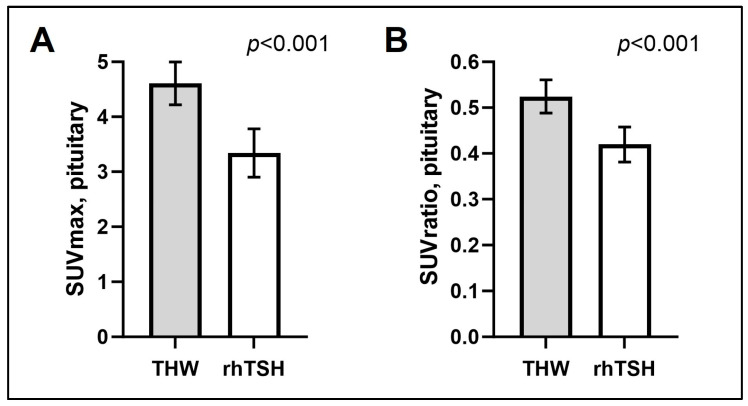
Comparison of pituitary SUVmax (**A**) and SUVratio (**B**) between the THW group and the rhTSH group. Data are shown as mean ±95% CI.

Compared to the rhTSH group, the THW group showed significantly higher cerebellum SUVmean values (mean ± SD: 8.91 ± 1.88, 95% CI: 9.31–9.51 vs. 7.99 ± 1.49, 95% CI: 7.23–8.76; *p* = 0.044), pituitary SUVmax values (mean ± SD: 4.61 ± 1.22, 95%CI: 4.22–5.00 vs. 3.34 ± 0.86, 95%CI: 2.9–3.8; *p* < 0.001), and SUVratio values (0.52 ±0.11, 95% CI: 0.49–0.56 vs. 0.42 ±0.07, 95% CI: 0.38–0.46; *p* < 0.001) (Figure 2). There was no difference in cerebellum SUVmean or in pituitary SUVmax or SUVratio values between men and women in either the THW or the rhTSH group.

**Figure 3:**
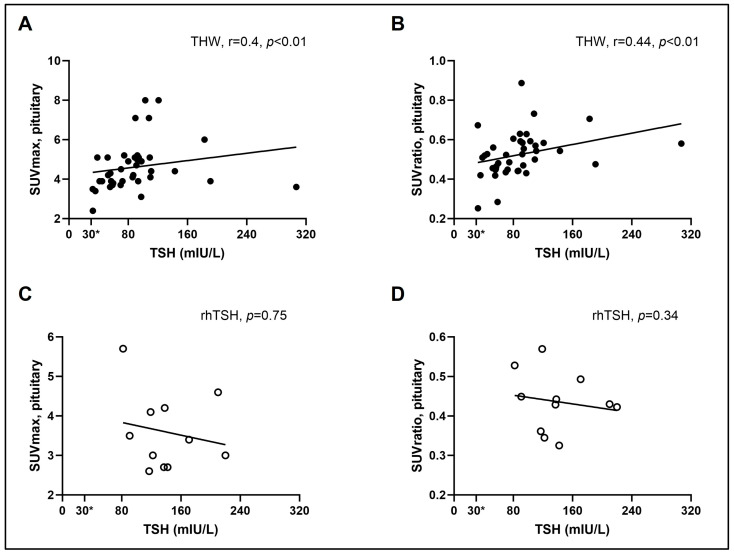
Spearman’s rank correlation tests between serum TSH levels and pituitary SUVmax or SUVratio in the THW (**A**,**B**) and rhTSH (**C**,**D**) groups. *, cutoff value of generally acceptable serum TSH levels for RAI therapy or diagnostic testing.

Finally, in the THW group, serum TSH levels correlated positively with the pituitary SUVmax (r = 0.41, *p* < 0.01) and SUVratio values (r = 0.44, *p* < 0.01), but not with the cerebellum SUVmean values; as expected, no correlations were present in the rhTSH group (Figure 3).

In the rhTSH group, pituitary SUV values were within normal ranges, except for four patients who had an SUVmax exceeding the threshold. In two of these cases, this could potentially be attributed to THW for 5–7 days before RAI administration (in addition to rhTSH administration), according to local clinical practice before 2016; serum-free T4 levels were below the reference range in one patient and low-normal in the other. Regarding the other two patients in the rhTSH group who had been treated without interruption of levothyroxine substitution and whose serum-free T4 levels were in the normal range, one was a woman with an SUVmax of 4.6, which could potentially be explained by her postmenopausal status and the associated stimulation of pituitary gonadotrope cells due to loss of negative feedback from estrogen [14]. However, there is no concrete evidence in the literature confirming pituitary hypermetabolism in postmenopausal women; on the contrary, a study demonstrating negative feedback of estrogen on ^18^FDG-PET/CT-assessed hypothalamic metabolic activity did not observe any change in pituitary metabolism [15]. Consistently, two-way ANOVA analysis of pituitary metabolism in the female patients of our cohort showed no main effect of menopausal status (premenopausal or postmenopausal without hormonal substitution therapy) (*p* = 0.82) and no interaction of menopausal status with the RAI preparation method (*p* = 0.23). The other patient was a man with an SUVmax of 4.1 and no obvious explanation for pituitary hypermetabolism. Nevertheless, the overall difference in pituitary metabolic activity between the THW and rhTSH groups was robust to these outliers.

## 4. Discussion

In the last decade, there has been a rapid rise in the use of ^18^F-FDG PET/CT for oncological and non-oncological indications. While incidental pituitary hypermetabolism is rare in the general population [7,8,9,10], the present study found that focally increased ^18^F-FDG uptake on PET/CT was present in 62.5% of patients with DTC under THW, which is even higher than the 52.4% frequency previously reported by Ding et al. [11]. The effects of thyroid hormones on the brain are complex, and previous studies have demonstrated that hypothyroidism affects different regional brain activities, with notably decreased activities in areas related to affect and cognition [16,17,18]. In addition, a study using ^18^F-FDG PET/CT to map metabolic brain networks in rats found that hypothyroidism induced fluctuations of brain network activity in the pituitary and hypothalamus, in accordance with the negative feedback mechanism; however, pituitary SUV values in euthyroid and hypothyroid rats were not specifically reported [19].

In patients with DTC, ^18^F-FDG PET/CT can be very useful to detect residual, recurrent, or metastatic disease, especially in the presence of elevated levels of serum thyroglobulin with negative RAI whole-body scan; stimulation by THW or rhTSH may improve the performance of ^18^F-FDG PET/CT scan in this context, in particular for low-volume disease [20]. Given that the previous study by Ding et al. included only patients with DTC under THW, we included here patients under rhTSH stimulation, most of whom were indeed biologically euthyroid. The mean SUVmax and mean SUVratio values were distinctively higher in the THW group compared to the rhTSH group (4.61 vs. 3.34 and 0.52 vs. 0.42, respectively), and values were in the same general range as those reported by Ding et al. in patients under THW vs. euthyroid subjects (interquartile ranges of 3.08–5.04 vs. 2.22–3.88 and 0.35–0.63 vs. 0.23–0.56, respectively) [11]. In contrast, studies reporting incidental pituitary ^18^F-FDG uptake associated with pituitary lesions showed a mean pituitary SUVmax of 15 [9], which is much higher than the values reported herein. Taken together, these observations strongly support that THW-associated pituitary hypermetabolism reflects a physiological response of the pituitary thyrotroph cells secondary to the loss/decrease in negative feedback inhibition of exogenous levothyroxine on the hypothalamic–pituitary–thyroid axis [21].

Regarding the reference region used for evaluating pituitary metabolic activity, the cerebellum SUVmean values were slightly but significantly higher in the THW group compared to the rhTSH group, whereas Ding et al. reported no significant difference in whole-brain SUVmean values between THW and euthyroid individuals (9.39 vs. 8.52; *p* = 0.37). Regardless of any regional differences in brain metabolic activity, the higher pituitary SUVratio values in THW patients cannot be attributed to the higher cerebellum SUVmean values since the latter are the denominator in the ratio.

It would be interesting to also quantify the hypothalamic metabolism, but due to the limited spatial resolution with PET/CT and the close proximity of the hypothalamus to other metabolically active brain areas, measurements of hypothalamic SUV were not possible in our study. Finally, it would be interesting to measure pituitary SUV following injection of TSH-releasing hormone (TRH), but the irradiating nature of PET/CT should be considered as an ethical limitation.

In this retrospective study, we did not include healthy subjects, but previous research has shown that the pituitary metabolic activity of patients with euthyroid DTC under levothyroxine substitution had no significant difference compared to healthy subjects [11]. In the present study, serum TSH levels of patients under THW correlated positively and strongly with SUVmax and SUVratio, similar to previous findings [11]. Conversely, no correlation was present in the rhTSH group, indicating that the increased levels of TSH were not the cause of pituitary hypermetabolism.

The characteristics of the cohort warrant some comments. The older age of patients in the rhTSH group compared to the THW group likely reflects real-life clinical practice, where hypothyroidism is best avoided in older and polymorbid patients for whom RAI treatment under rhTSH stimulation is generally preferred. For example, among 17 patients in the rhTSH group, 1 patient older than 80 years would normally have been treated with RAI under THW were it not for his age and general health status. Also, some of the patients in the rhTSH group had been imaged without RAI therapy. Finally, regarding sex, while the overall predominance of women was expected, the difference in the male/female ratio between the THW and rhTSH groups was not. This discrepancy could be explained by a selection bias in the rhTSH group, which included not only low and intermediate-risk DTC but also high-risk DTC with advanced age and significant comorbidities; indeed, previous studies have correlated male sex with a higher risk for advanced thyroid cancer [22,23]. Although the present work was not designed to address sexual dimorphism in the outcomes studied, there was no difference in pituitary SUVmax or SUVratio values between men and women in either the THW or the rhTSH group.

Lastly, this study has certain limitations due to its retrospective nature. Over the 8 years of patient recruitment, preparation protocols have been modified and modernized, most notably limiting the duration of THW to 3 weeks according to current concepts and practices [13]. Furthermore, the timing of ^18^F-FDG PET/CT scans in relation to RAI treatment (upon admission vs. some days later) was also variable, and laboratory thyroid function tests were not always collected at the moment of imaging but within 7 days of it. Nevertheless, the differences in pituitary metabolism between the THW and rhTSH groups were robust to these various aspects. Finally, even though the size of the cohort was small and did not allow for multivariate analyses, it exceeded the prespecified power and sample size calculation for the intended main comparison. Larger studies addressing the issue are warranted, as well as, ideally, studies examining the same patients under rhTSH and THW conditions on the same PET/CT instrument.

## 5. Conclusions

In conclusion, the commonly observed pituitary hypermetabolism in patients with DTC undergoing RAI treatment with THW should be considered physiological, likely reflecting the response of pituitary thyrotroph cells. When 18F-FDG PET/CT is performed with rhTSH stimulation, pituitary hypermetabolism is observed only in a minority of patients, and the SUV values are generally lower than those of patients with pituitary incidentalomas.

## Figures and Tables

**Table 1:** Comparison of patient characteristics, thyroid function tests, PET/CT acquisition parameters, tumor histology, and indication for PET/CT between the THW and rhTSH groups.

	THW (*n* = 40)	rhTSH (*n* = 17)	*p*-Value
Patient characteristics			
Age (years, mean ± SD)	43.26 ± 16.97	54.68 ± 15.36	0.02
Sex (male/female)	6/34	10/7	0.003
Weight (kg, mean ± SD)	71.5 ± 18.9	79 ± 21.33	0.19
BMI (kg/m^2^, mean ± SD)	28.85 ± 6.05	25.82 ± 5.24	0.99
**Thyroid function tests *^n^***			
Serum-free T4 (pmol/L, mean ±SD)	3.08 ± 1.42	14.17 ± 5.28	<0.0001
Serum-free T4 ≥12 pmol/L	0/43 (0%)	7/10 (70%)	<0.0001
Serum TSH (mIU/L, mean ±SD)	88.38 ± 50.37	140.8 ± 44.01	0.003
**PET-FDG acquisition parameters**			
^18^F-FDG activity (MBq, mean ±SD)	245 ± 51.32	261.8 ± 57.55	0.28
Glycemia (mmol/L, mean ±SD)	5.11 ± 0.95	5.07 ± 0.47	0.89
Time post-injection (min, mean ±SD)	65.93 ± 9	61.82 ± 6.79	0.10
**Tumor histology**			
Papillary	34/40 (85%)	12/17 (70.6%)	0.21 ^¥^
Classical	27/34 (79.4%)	8/12 (66.7%)	0.37 ^§^
Follicular variant	1/34 (2.9%)	3/12 (25%)	0.02 ^§^
Aggressive variant (tall cell, diffuse sclerosing, oncocytic, PDCT component)	6/34 (17.6%)	1/12 (8.3%)	0.44 ^§^
Follicular	2/40 (5%)	2/17 (11.8%)	0.36 ^¥^
Aggressive variant (widely invasive, PDTC component)	2/2 (100%)	1/2 (50%)	
PDTC	2/40 (5%)	0/17 (0%)	0.35 ^¥^
Oncocytic	2/40 (5%)	3/17 (17.6%)	0.12 ^¥^
Widely invasive	2/2 (100%)	2/3 (66.7%)	
Multifocality	19/40 (47.5%)	6/17 (35.3%)	0.06
Lymph node metastasis	28/40 (70%)	10/17 (58.8%)	0.41
Extrathyroidal extension (T3b-T4)	7/40 (17.5%)	1/17 (5.9%)	0.25
Distance metastasis	3/40 (7.5%)	0/17 (0%)	0.25
**PET/CT indication**			
Suspicion of iode-refractory disease	12/40 (30%)	8/17 (47.1%)	0.22
Advanced initial presentation	24/40 (60%)	7/17 (41.2%)	0.19
Extrathyroidal extension (ETE)	10/24 (41.7%)	5/7 (71.4%)	0.17
Gross ETE	3/10 (30%)	0/5 (0%)	
Minimal ETE	7/10 (70%) *	5/5 (100%) ^#^	
Lymph node metastasis	22/24 (91.7%)	7/7 (100%)	0.43
Aggressive histology	11/40 (27.5%)	5/17 (29.4%)	0.88

PDTC, poorly differentiated thyroid carcinoma; BMI, body mass index; *n*: in the rhTSH group, free T4 values were available in 10/17 patients and TSH values in 11/17 patients; ¥, chi-square between histology subtypes; §, chi-square between papillary variants; *, 6/7 had lymph node metastasis; #, all 5 had lymph node metastasis.

## Data Availability

The data associated with this study are included in Supplementary Table S1, available online (https://doi.org/10.5281/zenodo.10782396 (accessed on 5 March 2024)).

## Supplementary Materials

The following supporting information can be downloaded at: https://www.mdpi.com/article/10.3390/cancers16071382/s1.
